# Global trends in phage-derived depolymerase research for anti-infective applications: a bibliometric analysis

**DOI:** 10.3389/fmicb.2026.1947768

**Published:** 2026-10-08

**Authors:** Guanglin Li, Junhui Li, Xiao Ma, Peipei Zhang, Jiye Zhang

**Affiliations:** 1 School of Pharmacy, Health Science Center, Xi'an Jiaotong University, Xi'an, China; 2 School of Computer Science and Engineering, Xi'an University of Technology, Xi'an, China; 3 Yunnan Botanee Bio-Technology Group Co., Ltd., Kunming, China

**Keywords:** anti-infective, bacteriophage, bibliometric analysis, biofilm, *Klebsiella pneumoniae*, phage-derived depolymerase

## Abstract

**Introduction:**

Phage-derived depolymerases, primarily located in phage tail fiber and tail spike proteins, have emerged as promising antibacterial and antivirulence agents capable of degrading bacterial surface polysaccharides and thereby combating multidrug-resistant bacterial infections. Despite the growing volume of publications in this interdisciplinary domain, a systematic bibliometric synthesis of the global research landscape remains absent.

**Methods:**

Publications indexed from 2010 to 2025 were retrieved from the Web of Science Core Collection (WOSCC) and Scopus on April 26, 2026. After deduplication and relevance screening, 326 records were retained. Bibliometric analyses and visualizations were performed using R with the bibliometrix package, VOSviewer and CiteSpace.

**Results:**

Annual publication output increased from 3 records in 2010 to 51 in 2025 and fitted an exponential growth model, indicating sustained expansion of the field. China and the United States were the most productive countries/regions, whereas Belgium and Poland showed the highest citation impact among leading contributors. The University of Wrocław and the Russian Academy of Sciences emerged as prominent institutional hubs. Keyword co-occurrence analysis identified three major thematic directions: phage discovery and genomic characterization, antibacterial and antibiofilm applications, and mechanistic or structural studies of depolymerase activity, with *Klebsiella pneumoniae* being the most frequently studied bacterial target.

**Conclusion:**

This bibliometric analysis systematically maps the 16-year landscape of phage depolymerase-enabled anti-infective research, delineating the leading forces and thematic evolution that have shaped the field. These trends highlight promising avenues for mechanistic studies on the structural basis of depolymerase substrate specificity and preclinical research on formulation, safety, and manufacturing, supporting the translational development of depolymerase-related anti-infectives.

## 1 Introduction

Bacterial infections remain a major global health challenge, particularly in the context of rapidly increasing antimicrobial resistance (AMR). A recent global burden analysis estimated that bacterial AMR was associated with 4.71 million deaths in 2021 and could contribute to as many as 8.22 million deaths annually by 2050 in the absence of effective interventions (Naghavi et al., 2024). The rising prevalence of multidrug-resistant (MDR) pathogens has eroded the clinical utility of many conventional antibiotics, underscoring the urgent need for novel antibacterial modalities (Murray et al., 2022). In response, the World Health Organization (WHO) has repeatedly emphasized the necessity of prioritizing research and development targeting drug-resistant bacterial pathogens (Tacconelli et al., 2018; Abdallah et al., 2026; Sati et al., 2025).

Natural microbial antagonism has long informed the discovery of antibacterial agents. Bacteriophages (phages)–the natural predators of bacteria–have regained substantial attention as potential alternatives or adjuncts to antibiotics, particularly for MDR infections. Encouraging progress in experimental and clinical studies has highlighted the therapeutic efficacy of phage-based strategies (Walter et al., 2023; Dagli et al., 2024; Zhou et al., 2026; Kim et al., 2025). Nevertheless, broader clinical implementation still faces several challenges, including the emergence of phage resistance, formulation and delivery constraints, pharmacokinetic complexity, and regulatory considerations (Siopi et al., 2024; Nguyen et al., 2026; Venkataraman et al., 2025). Continuous bacteria-phage co-evolution drives the emergence of diverse bacterial antiphage defense systems–including receptor modification, restriction-modification, CRISPR-Cas systems, and abortive infection–which may influence the long-term efficacy of intact virion therapy (Karamlou et al., 2025; Labrie et al., 2010).

In this context, phage-encoded polysaccharide depolymerases constitute a more chemically defined class of antibacterial and antivirulence effectors (Wang et al., 2024a). These enzymes need not be directly bactericidal; instead, they are commonly present as domains within tail fiber proteins (TFPs) or tail spike proteins (TSPs) that mediate receptor recognition and polysaccharide cleavage (Islam et al., 2024; Zhang et al., 2025c). They target capsular polysaccharides (CPS, also known as K antigens), exopolysaccharides (EPS), or lipopolysaccharide (LPS) O-antigen glycans, thereby disrupting protective surface barriers, attenuating virulence, impairing biofilm integrity, and sensitizing bacteria to host immunity and antibiotics (Oliveira et al., 2019b; Liu et al., 2019a; Maciejewska et al., 2023; Chen et al., 2022; Zhang et al., 2025a; Leiman et al., 2007; Oliveira et al., 2019a). Because depolymerases primarily target non-essential surface antigens, they may impose a weaker selective pressure for resistance than conventional bactericidal agents (Lin et al., 2018). Beyond their biological activity, depolymerases also possess several translational advantages. As purified recombinant proteins, depolymerases are amenable to scalable production, standardized formulation, and rational protein engineering–features that facilitate manufacturing and quality control relative to whole-phage biologics (Wang et al., 2024a). These properties have promoted the exploration of depolymerases as complementary or even standalone therapeutic agents in diverse applications beyond direct antibacterial effects, including antibiofilm interventions, antivirulence strategies, synergistic antibiotic combinations, and immune-assisted clearance.

As the therapeutic potential of polysaccharide depolymerases in anti-infective applications has become increasingly evident, the volume of related publications has grown steadily. Accordingly, systematic approaches are increasingly needed to map research trends, synthesize emerging evidence, and identify major developments within this expanding field. While narrative reviews have provided valuable qualitative summaries based on expert-selected evidence (Guo et al., 2023; Islam et al., 2024; Oliveira et al., 2022; Topka-Bielecka et al., 2021; Pires et al., 2016), bibliometric analysis offers a complementary quantitative framework that leverages systematic publication data to map the field's structural backbone and temporal evolution, thereby extending and refining expert syntheses. Specifically, by applying mathematical and statistical tools to large publication datasets, bibliometrics enables researchers to efficiently identify leading authors, institutions, collaborative networks, and emerging research themes within a specific domain (Xie et al., 2024; Zhang et al., 2025b; Song et al., 2026; Abdelhadi et al., 2026). Such analyses can further facilitate knowledge dissemination and help reveal developmental trajectories and future directions in the field.

Against this background, the present study employs bibliometric methods to systematically map the global research landscape of phage-derived polysaccharide depolymerases toward anti-infective therapy from 2010 to 2025, covering publication trends, countries/regions, institutions, authors, journals, keywords, and references. This analysis aims to identify major research contributors, characterize areas of concentrated scientific activity, and evaluate evolutionary trajectory and emerging frontiers. By providing a comprehensive quantitative overview and thoughtful interpretation, this study may help researchers better contextualize current progress and identify priorities for future research and translational development of phage-derived depolymerase-based anti-infective strategies.

## 2 Methods

### 2.1 Data collection and screening

Literature data were retrieved from the Web of Science Core Collection (WOSCC) and Scopus. WOSCC served as the primary source because of its mature citation indexing and interoperability with bibliometric workflows, whereas Scopus was queried in parallel to broaden coverage of titles and abstracts and to reduce single-database omission bias. Boolean queries with database-specific field tags were employed to ensure reproducible retrieval. In WOSCC, the search strategy was: TS = (bacteriophage* OR phage*) AND TS = (depolymerase* OR “tail fiber protein” OR “tail spike protein” OR “tailspike protein”) AND TS = (antibacterial OR antimicrobial OR antibiofilm OR therapeutic OR treatment). The corresponding Scopus query was: TITLE-ABS-KEY (bacteriophage* OR phage*) AND TITLE-ABS-KEY (depolymerase* OR “tail fiber protein” OR “tail spike protein” OR “tailspike protein”) AND TITLE-ABS-KEY (antibacterial OR antimicrobial OR antibiofilm OR therapeutic OR treatment). The publication window was set from January 1, 2010, through December 31, 2025. Years prior to 2010 were excluded due to sparse and intermittent annual records (several years missing, most with fewer than two papers per year), which lack statistical robustness for trend analysis. The search was restricted to English-language original articles and reviews, and all searches were finalized on April 26, 2026.

A three-step screening process was applied. First, records that were not English-language original articles or reviews were excluded during database retrieval. Second, after merging the WOSCC and Scopus records, duplicates were removed using the bibliometrix package in R (version 4.5.3). Third, titles and abstracts were independently screened for relevance by two reviewers (GL and XM), with discrepancies resolved by consensus. The exclusion criteria for this relevance screening were as follows: (1) studies that only involved phage genome sequencing, biological characterization, or lytic activity, without addressing phage-encoded polysaccharide depolymerases (including putative depolymerases), polysaccharide-degrading or antibiofilm tail fiber/spike proteins (RBPs, TSPs, and TFPs), or depolymerase-related host recognition spectra; and (2) studies that only involved phage-encoded endolysins, without combining or comparing them with polysaccharide depolymerases. These criteria were deliberately kept broad to maximize retrieval sensitivity and avoid prematurely excluding early foundational studies and extended research related to phage depolymerase anti-infective applications. Finally, an analytical corpus of 326 publications (274 articles and 52 reviews) was obtained. The screening workflow is summarized in Figure 1.

**Figure 1:**
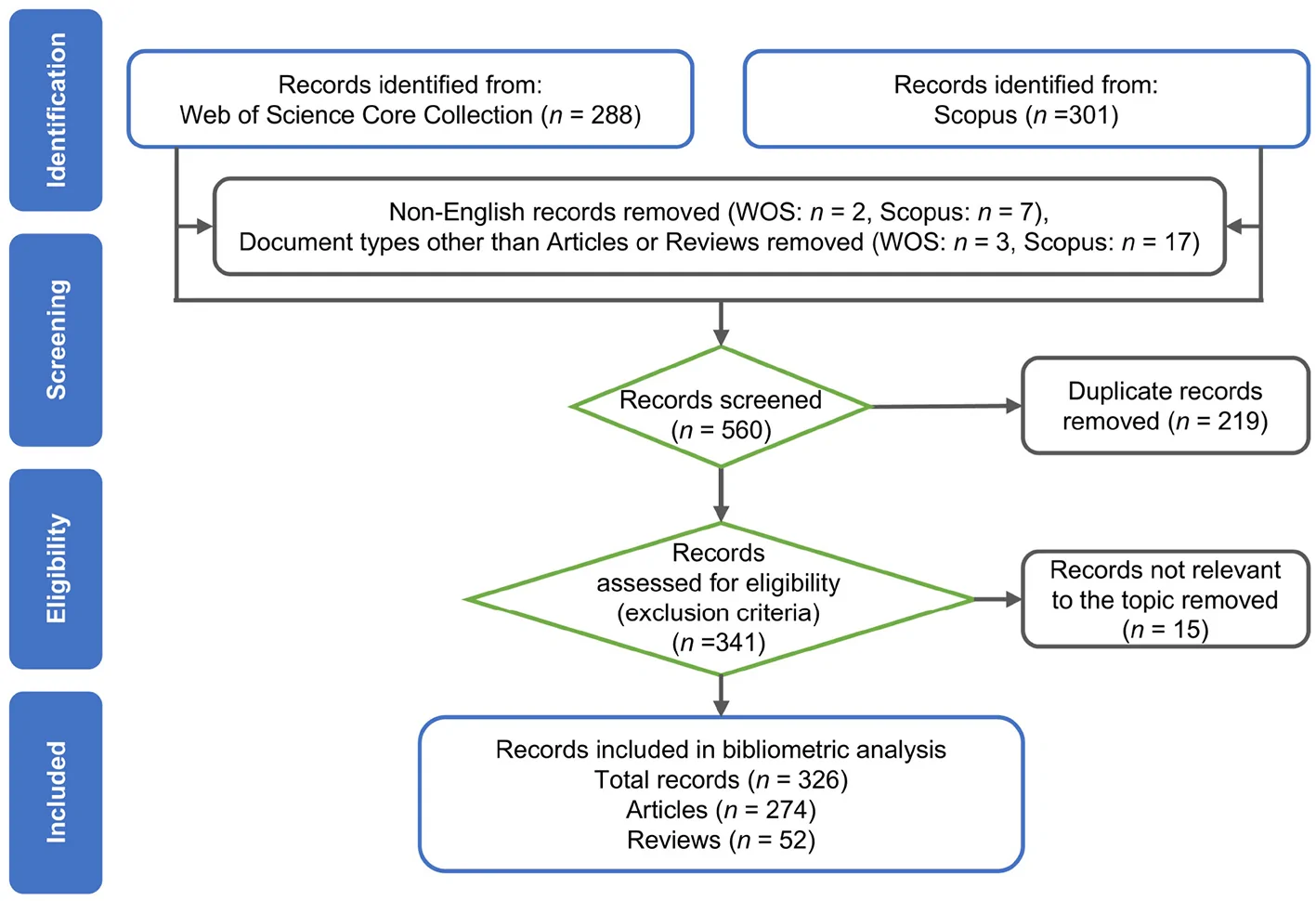
Flowchart of literature retrieval and screening process.

### 2.2 Bibliometric analysis

Data preprocessing, bibliometric analyses, and visualizations were conducted using R with the bibliometrix package, VOSviewer (version 1.6.20) and CiteSpace (version 6.4 R1). Countries/regions, institutions, and author names were analyzed according to the standardized formats exported directly from the databases. Descriptive bibliometric analyses, including summaries of annual publication output, citation metrics, and the productivity of countries/regions, institutions, authors, journals and keywords were conducted via the bibliometrix R package. Temporal distribution of countries/regions, journals and keywords were also carried out in R. Three classical bibliometric laws were applied to characterize the structure of the literature–Price's law was used to model the exponential growth trend of annual publication output (Price, 1976), Lotka's law to evaluate the distribution of author productivity (Kushairi and Ahmi, 2021), and Bradford's law to identify the core journals in this field (Nash-Stewart et al., 2012). Annual citation counts were modeled using a negative binomial generalized additive model (NB-GAM) to account for the count nature and overdispersion of citation data (Thurston et al., 2000). Collaboration networks (countries/regions, institutions, authors), journal co-citation networks and keyword co-occurrence maps were constructed using VOSviewer. Co-cited references network and timeline map, as well as citation bursts were generated using CiteSpace.

## 3 Results

### 3.1 Annual publication and citation trends

After systematic retrieval and deduplication, 326 publications (274 original articles and 52 reviews) were retained for bibliometric analysis (Figure 1). As shown in Figure 2, annual publications related to phage-derived depolymerases in anti-infective research exhibited an overall increasing trend from 2010 to 2025. Output rose from 3 publications in 2010 to 51 in 2025–approximately a 17-fold increase across the study interval. Growth was comparatively moderate before 2017 and accelerated markedly after 2020, consistent with intensified global interest in depolymerase-enabled anti-infective strategies. According to Price's law, the annual publication output conformed to an exponential growth model described by *y* = 4.18*e*^0.1732(*x*−2010)^, with a strong fitting coefficient (*R*^2^ = 0.939). Annual citation totals fluctuated across years but showed an overall increasing trend, with the first derivative of the fitted GAM remaining positive throughout the study period (Supplementary Figure S1). The relatively lower citation counts observed for the most recent years likely reflect shorter citation accumulation windows rather than diminished scholarly impact. These findings indicate that phage depolymerase-related anti-infective research has attracted growing academic attention and has entered a stage of accelerated growth in recent years.

**Figure 2:**
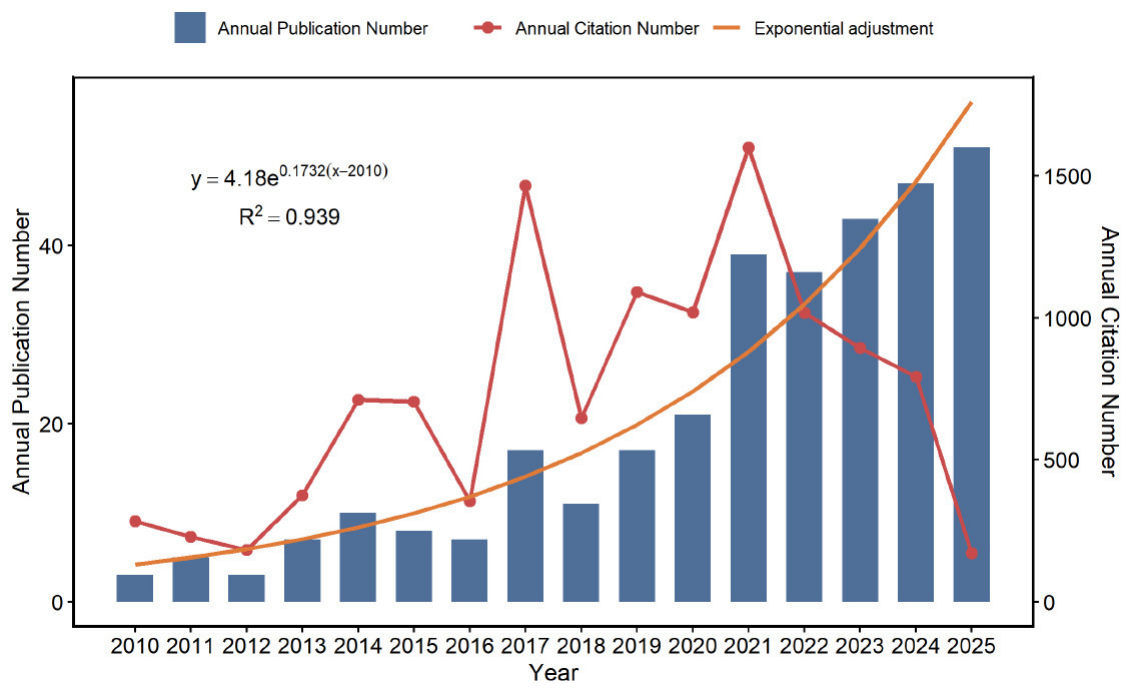
Annual publication and citation trends in phage-derived depolymerase research. Blue bars represent annual publication output, red lines indicate annual citation counts, the orange curve represents the exponential fitting trend based on Price's law.

### 3.2 Distribution of productive countries/regions and academic collaboration

A total of 54 countries/regions contributed to phage depolymerase-related anti-infective research. China led in publication volume (*n* = 97) and the highest H-index (29), followed by the United States (USA, *n* = 58, H-index = 28), whereas the USA exhibited a substantially higher CPP (citations per publication, 47.07) than China (22.37) (Table 1 and Figure 3A). Although China had the highest output, its overall CPP was lower than that of most top 10 countries/regions. We interpret this qualitatively as being due to its recent output surge shortening the citation window, rather than as a formal time-normalized conclusion; its recent annual CPP was similar to that of other countries (Supplementary Figure S2). Belgium and Poland showed the highest CPPs (89.79 and 76.85, respectively, Table 1 and Figure 3A). Annual trends (Figure 3B) show China's clear dominance, with marked growth after 2018, surpassing the USA in 2022 and expanding thereafter, while the USA maintained stable second-place output. Geographically, publications concentrated in Asia, North America, and Europe (Figure 3C). In terms of collaboration, the USA ranked first in multiple-country publications (MCP) count (*n* = 28), followed by China (*n* = 25) and England (*n* = 15), while China's output was predominantly single-country publication (SCP)-driven, indicating domestic research strength. The USA also had the highest total link strength (TLS, 56), with Belgium (35), England (34), and Poland (30) as key collaborative nodes (Figure 3D, Table 1). Overall, the field has been propelled by both concentrated national productivity and active international cooperation.

**Table 1:** Top 10 most productive countries/regions in phage depolymerase-related anti-infective research.

Rank	Country/region	No. of publications	MCP	SCP	No. of citations	CPP	H-index	TLS
1	China	97	25	72	2170	22.37	29	28
2	United States	58	28	30	2730	47.07	28	56
3	Russia	34	5	29	714	21.00	13	9
4	Poland	26	12	14	1998	76.85	20	30
5	England	18	15	3	644	35.78	12	34
6	Taiwan	15	3	12	845	56.33	12	3
7	Portugal	15	9	6	743	49.53	11	17
8	India	15	4	11	477	31.80	10	7
9	Belgium	14	14	0	1257	89.79	13	35
10	Spain	14	6	8	388	27.71	10	7

MCP, multiple-country publication; SCP, single-country publication; CPP, citations per publication; TLS, total link strength.

**Figure 3:**
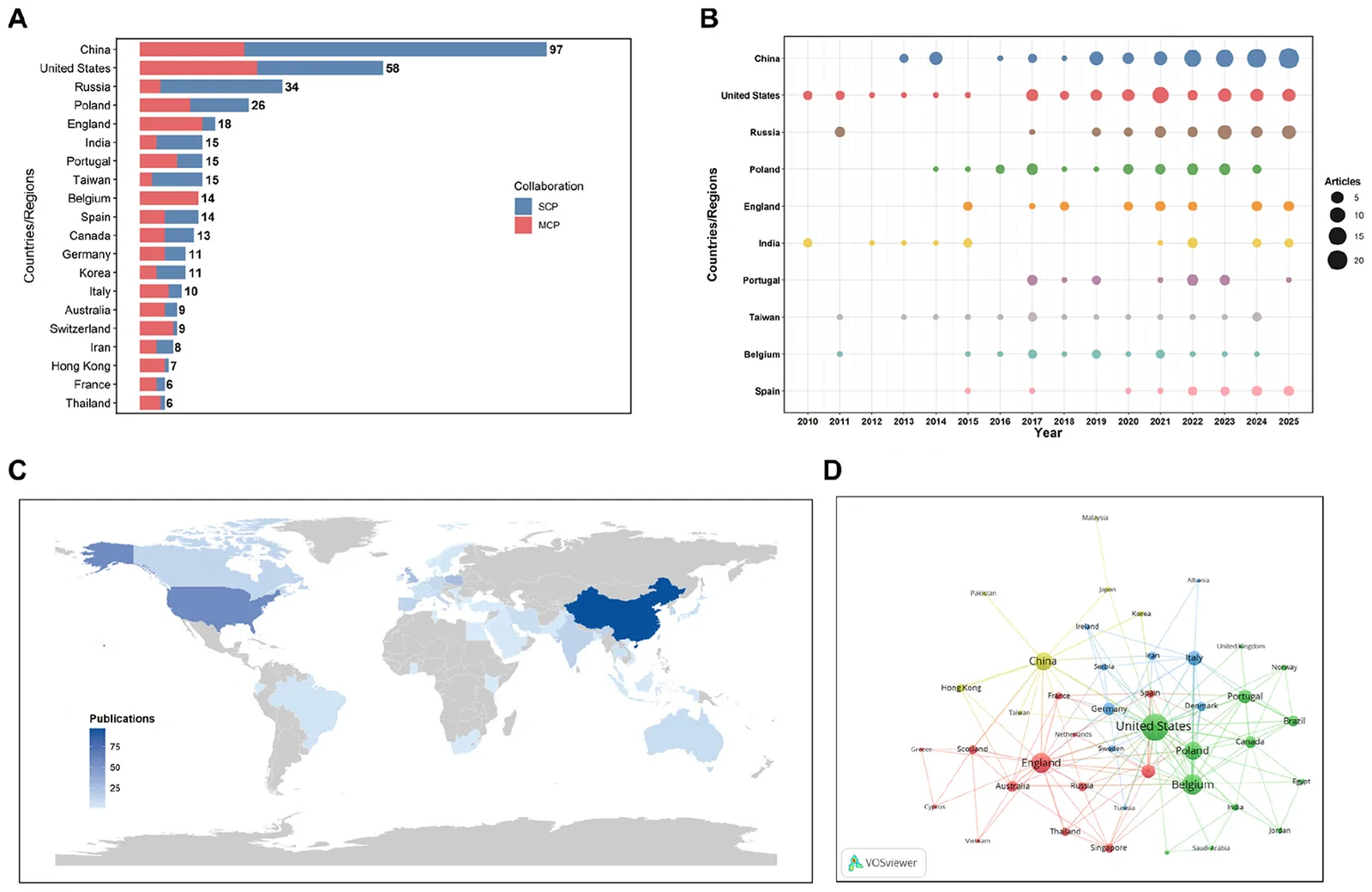
Distribution and collaboration network of the most productive countries/regions in phage depolymerase-related anti-infective research. **(A)** Publication output of the top 20 most productive countries/regions, classified as single-country publications (SCP) and multiple-country publications (MCP). **(B)** Annual publication trends of the top 10 most productive countries/regions from 2010 to 2025. Bubble size represents the annual number of publications. **(C)** Global geographic distribution of publications by country/region. Darker colors indicate higher publication output. **(D)** International collaboration network of countries/regions generated by VOSviewer, which includes 42 countries/regions with a total link strength (TLS) > 2. Node size represents the number of publications by each country/region, links indicate collaborative relationships among countries/regions, and different colors represent distinct country/region clusters.

### 3.3 Most productive institutions and collaborative networks

During the study period, 531 institutions participated in this research area. Table 2 and Figure 4A profiles the ten most prolific institutions, which collectively accounted for 71 unique publications (21.78% of the analytical corpus). The Russian Academy of Sciences led in publication count (*n* = 16), followed by the University of Wrocław (*n* = 15) and Nanjing Agricultural University (*n* = 13). In terms of citation impact, the University of Wrocław ranked first (1477 citations, CPP = 86.88), followed by Catholic University of Leuven (Katholieke Universiteit Leuven, KU Leuven, 644 citations, CPP = 71.56) and National Taiwan University (567 citations, CPP = 63.00). The institutional collaboration network (Figure 4B) revealed six major clusters, led by the Russian Academy of Sciences as the central node and the University of Wrocław, along with other key institutions such as Spanish National Research Council (Consejo Superior de Investigaciones Científicas, CSIC), KU Leuven, the University of London, and Ghent University. Overall, the leading institutions exhibited strong research productivity and collaborative connectivity, highlighting the important role of established research centers in advancing this field.

**Table 2:** Top 10 most productive institutions in phage depolymerase-related anti-infective research.

Rank	Institution	No. of publications	No. of citations	CPP	H-index	TLS
1	University of Wrocław	17	1477	86.88	15	57
2	Russian Academy of Sciences	16	343	21.44	10	56
3	Nanjing Agricultural University	15	341	22.73	10	45
4	CSIC	12	359	29.92	10	44
5	University of Minho	10	518	51.80	7	26
6	KU Leuven	9	644	71.56	8	43
7	National Taiwan University	9	567	63.00	9	24
8	Zelinsky Institute of Organic Chemistry	9	276	30.67	9	36
9	Lomonosov Moscow State University	8	184	23.00	6	24
10	University of Valencia	8	169	21.12	5	27

CPP, citations per publication; TLS, total link strength.

**Figure 4:**
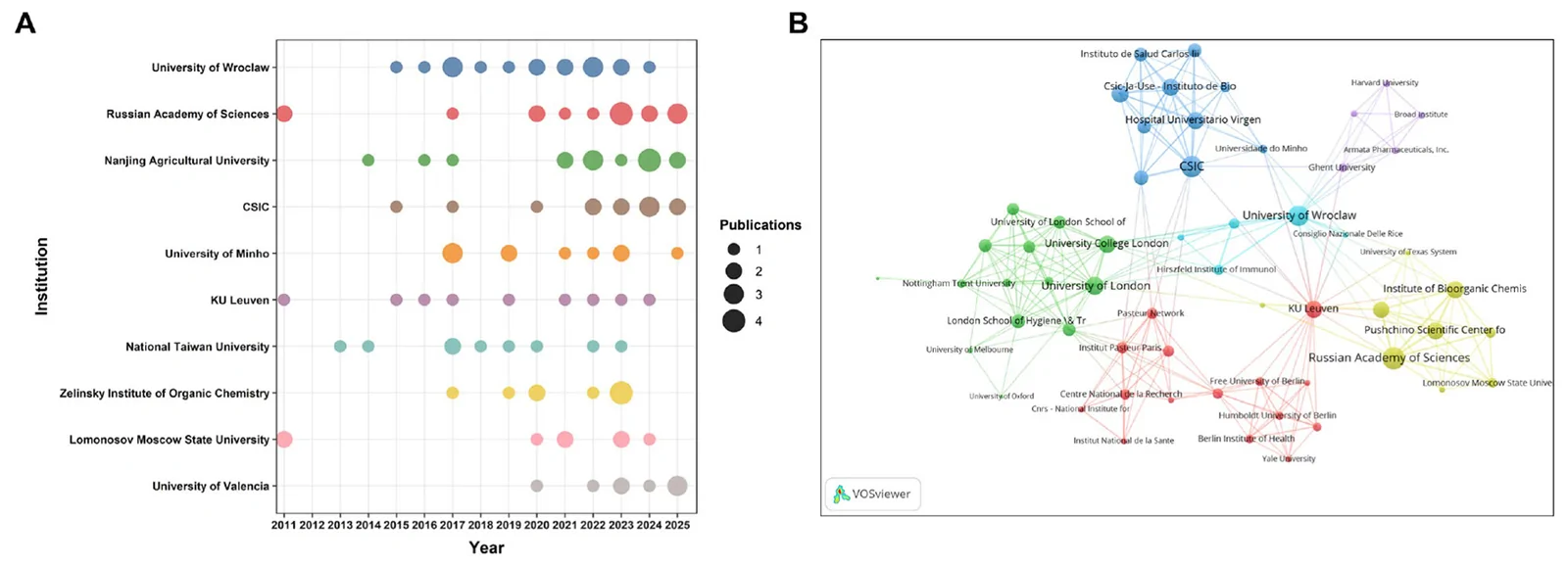
Institution productivity and collaboration network in phage depolymerase-related anti-infective research. **(A)** Annual publication trends of the top 10 most productive institutions from 2010 to 2025. Bubble size represents the annual number of publications. **(B)** Institutional collaboration network was generated by VOSviewer and includes institutions with a total link strength (TLS) > 10. Node size represents the number of publications by each institution, links indicate collaborative relationships among institutions, and different colors represent distinct institution clusters.

### 3.4 Most prolific authors and core co-authorship analysis

A total of 1,779 authors contributed to the publications. The author productivity distribution conformed to Lotka's law, with a strong power-law fit (*R*^2^ = 0.9552, β = 2.8304), and the Kolmogorov-Smirnov test confirmed no significant deviation from the theoretical distribution (*p* = 0.0745, *p* > 0.05) (Figure 5A). The elevated β value indicates greater productivity concentration than the classical β = 2 model. It is worth noting that the classical exponent β = 2 is itself a special case, as empirical β estimates often vary considerably across disciplines (Vlachý, 1978; Sahu and Jena, 2022). Table 3 lists the top 10 most productive authors. Drulis-Kawa Zuzanna ranked first (*n* = 16), followed by Zhang Wei (*n* = 14) and Knirel Yuriy A (*n* = 10). Maciejewska Barbara achieved the highest CPP (132.62 across 8 publications). Regarding collaboration strength, Zhang Wei exhibited the highest TLS value (125), followed by Knirel Yuriy A (115), Drulis-Kawa Zuzanna (108) and Shneider Mikhail M (108). The core co-authorship network (Figure 5B) included 36 authors forming four distinct clusters. The red cluster was the largest and centered on Volozhantsev Nikolay V from the State Research Center for Applied Microbiology and Biotechnology. The green cluster was primarily centered on Knirel Yuriy A et al. from the Russian Academy of Sciences. The blue cluster was mainly associated with Drulis-Kawa Zuzanna et al. from the University of Wrocław and Lavigne Rob from KU Leuven, while the yellow cluster predominantly comprised Oliveira Hugo et al. from the University of Minho. The green and blue clusters bridged the red and yellow clusters, indicating their intermediary roles. Notably, the two authors from Nanjing Agricultural University in the top 10 had relatively high TLS values but did not appear in this core co-authorship network (Table 3 and Figure 5B). Together with China's high SCP proportion, lower MCP/SCP ratio, and country-level TLS that was only about half that of the USA, this pattern suggested that Chinese researchers were more likely to engage in domestic or institution-centered collaborations rather than broader cross-national collaborative networks (Table 1, Figures 3A, D, 4B). Overall, the gradual emergence of stable teams and mature collaborations has become central to advancing phage depolymerase-related anti-infective research.

**Figure 5:**
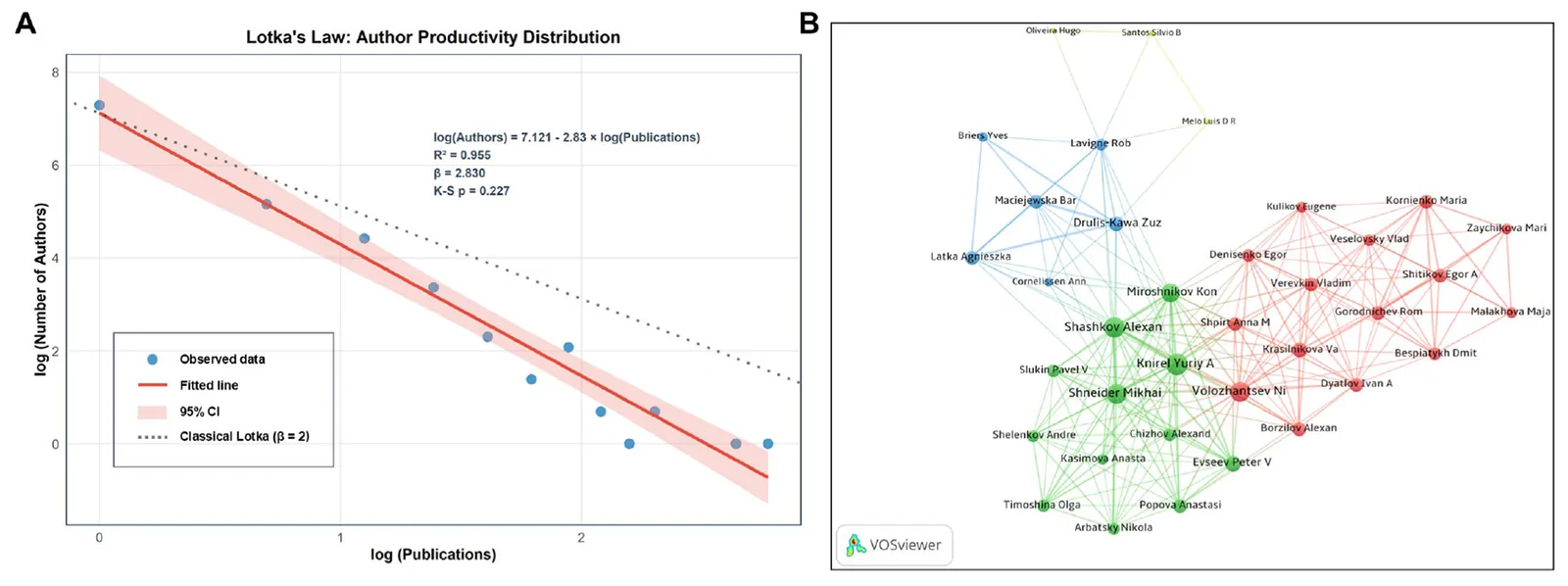
Author productivity and collaboration patterns in phage depolymerase-related anti-infective research. **(A)** Lotka's law distribution of author productivity. The scatter plot presents the log–log relationship between the number of publications per author and the corresponding number of authors. Each blue dot represents the observed frequency of authors with a given publication count. The red solid line indicates the fitted power-law model, with the light red shaded area denoting the 95% confidence interval (CI). The gray dotted line represents the classical Lotka's law reference (β = 2). The Kolmogorov-Smirnov goodness-of-fit test (*p* > 0.05) confirms that the observed distribution is statistically consistent with the theoretical Lotka model. **(B)** Author co-authorship network. The network was generated by VOSviewer and includes authors with a total link strength (TLS) >25. Node size represents the number of publications by each author, links indicate collaborative relationships among authors, and different colors represent distinct author clusters.

**Table 3:** Top 10 most productive authors in phage depolymerase-related anti-infective research.

Rank	Author	Institution	No. of publications	No. of citations	CPP	H-index	TLS
1	Drulis-Kawa Zuzanna	University of Wrocław	16	1454	90.88	14	108
2	Zhang Wei	Nanjing Agricultural University	14	315	22.50	10	125
3	Knirel Yuriy A	Russian Academy of Sciences	10	294	29.40	9	115
4	Li Pei	Nanjing Agricultural University	10	195	19.50	8	100
5	Shneider Mikhail M	Russian Academy of Sciences	9	222	24.67	8	108
6	Maciejewska Barbara	University of Wrocław	8	1061	132.62	8	56
7	Shashkov Alexander S	Russian Academy of Sciences	8	263	32.88	8	94
8	Łątka Agnieszka	University of Wrocław	7	745	106.43	7	58
9	Lavigne Rob	KU Leuven	7	579	82.71	6	68
10	Lin Tzu-Lung	National Taiwan University	7	572	81.71	7	54

CPP, citations per publication; TLS, total link strength.

### 3.5 Source journals and journal co-citation analysis

A total of 133 journals published articles in this field. Bradford zone analysis (Table 4) identified 7 core journals (5.3% of all journals) contributing 110 articles (33.7% of publications), confirming a concentration consistent with Bradford's law (Nash-Stewart et al., 2012; Rao, 2006). Among the top 10 most productive journals (Table 5), *Frontiers in Microbiology* ranked first (*n* = 30), followed by *Viruses-Basel* (*n* = 21) and *Antibiotics-Basel* (*n* = 15). Notably, *PLOS ONE* ranked 8th in output but achieved the highest CPP (78.88) among top 10, while *Cell* contributed only one article yet received 331 citations, indicating occasional high-impact multidisciplinary contributions (Supplementary Figure S3). Annual publication trends (Figure 6A) show that *Frontiers in Microbiology, Viruses-Basel, Antibiotics-Basel*, and *International Journal of Molecular Sciences* remained consistently active from 2019 onward. Journal co-citation analysis (Figure 6B) identified *Frontiers in Microbiology, PLOS ONE, Viruses-Basel, Applied and Environmental Microbiology, Antimicrobial Agents and Chemotherapy*, and *Applied Microbiology and Biotechnology* as key highly co-cited nodes. Overall, this field is predominantly supported by specialized microbiology and virology journals, with additional contributions from selected multidisciplinary high-impact journals.

**Table 4:** Bradford's law distribution of journals in phage depolymerase-related anti-infective research.

Zone	No. of publications (percentage)	Cumulative publications (percentage)	No. of journals (percentage)	Cumulative journals (percentage)
I	110 (33.7%)	110 (33.7%)	7 (5.3%)	7 (5.3%)
II	109 (33.4%)	219 (67.2%)	30 (22.6%)	37 (27.8%)
III	107 (32.8%)	326 (100%)	96 (72.2%)	133 (100%)

Percentage, proportion of total publications or journals included in this study.

**Table 5:** Top 10 most productive journals in phage depolymerase-related anti-infective research.

Rank	Journal	No. of publications (percentage)	No. of citations	CPP	H-index	JCR Q	JIF
1	*Frontiers in Microbiology*	30 (9.20%)	1179	39.30	19	Q1	5.8
2	*Viruses-Basel*	21 (6.44%)	660	31.43	15	Q2	3.8
3	*Antibiotics-Basel*	15 (4.60%)	694	46.27	13	Q1	5.5
4	*Scientific Reports*	12 (3.68%)	619	51.58	10	Q1	4.9
5	*Microorganisms*	11 (3.37%)	217	19.73	8	Q2	4.7
6	*International Journal of Molecular Sciences*	11 (3.37%)	150	13.64	6	Q1	5.6
7	*Microbiology Spectrum*	10 (3.07%)	160	16.00	6	Q2	4.1
8	*PLOS ONE*	8 (2.45%)	631	78.88	8	Q2	2.8
9	*Applied and Environmental Microbiology*	7 (2.15%)	321	45.86	6	Q2	4.2
10	*Frontiers in Cellular and Infection Microbiology*	7 (2.15%)	190	27.14	6	Q1	5.5

CPP, citations per publication; percentage, proportion of total publications included in this study; JCR Q, Journal Citation Reports quartile (2026 release); JIF, 2025 Journal Impact Factor (2026 release).

**Figure 6:**
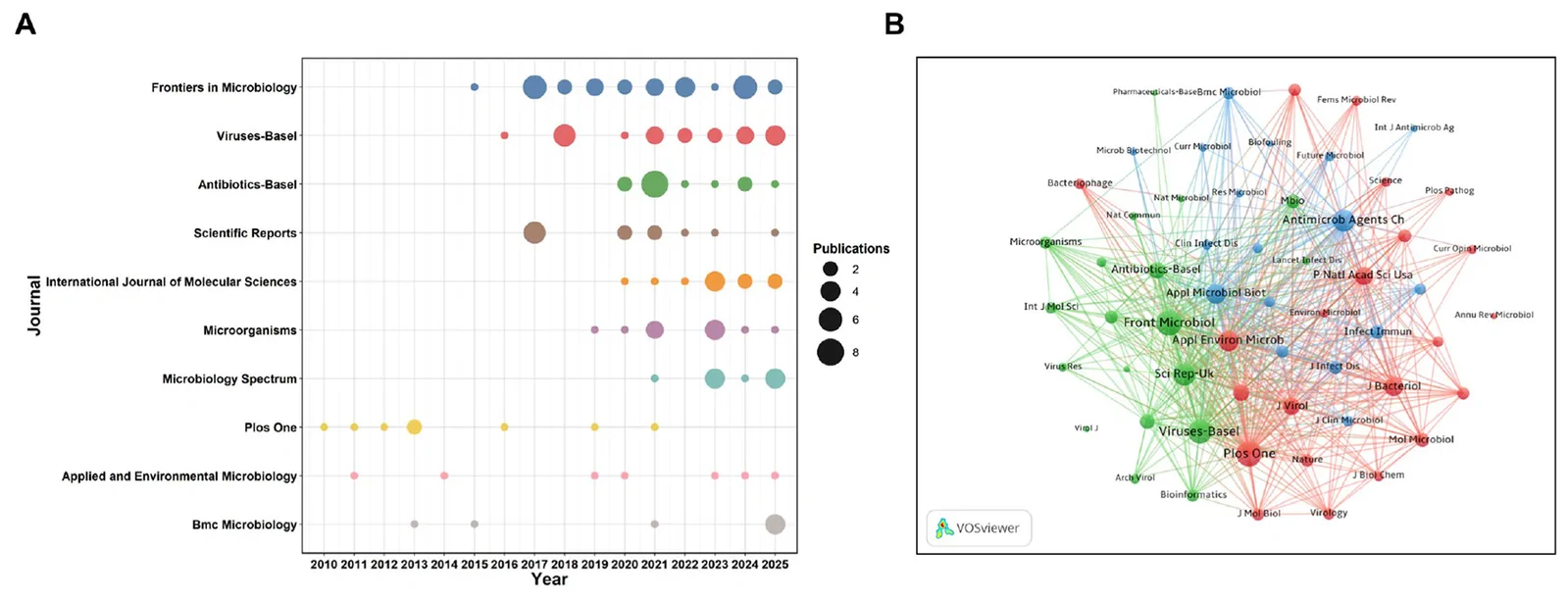
Journal productivity and journal co-citation network in phage depolymerase-related anti-infective research. **(A)** Annual publication trends of the top 10 most productive journals from 2010 to 2025. Bubble size represents the annual number of publications. **(B)** Journal co-citation network generated by VOSviewer with a total link strength (TLS) > 2,500. Node size represents the co-citation frequency of journals, links indicate co-citation relationships, and different colors represent distinct journal clusters.

### 3.6 Co-cited references analysis

A co-cited reference network was constructed using CiteSpace (Figure 7A), including 9833 cited references. The network showed a modularity Q of 0.8474 and a weighted mean silhouette of 0.9433, indicating a well-structured clustering result. Among the top 10 clusters, “therapeutic potential” and “pathogen detection” were the two largest and most densely connected clusters, highlighting the dual focus on therapeutic and diagnostic applications. The timeline visualization (Figure 7B) reveals that earlier studies concentrated on pathogen detection, while recent research has increasingly shifted toward therapeutic potential. Citation burst analysis (Figure 7C) identified 25 burst references between 2019 and 2025, with the strongest citation bursts highlighted as follows. The review by Knecht et al. (2020) on the diversity and function of phage-encoded depolymerases exhibited the strongest citation burst (strength = 13.99), underscoring its high influence in the field. Among the burst references, 16 involved the identification and characterization of polysaccharide depolymerases, of which 8 reported the therapeutic efficacy of these enzymes in mouse or *Galleria mellonella* infection models. This reflects that novel substrate specificity and anti-infective activity of depolymerases are key research focus in the field. Remarkably, 18 out of the 25 burst references remained active through 2025, indicating their sustained influence on current research.

**Figure 7:**
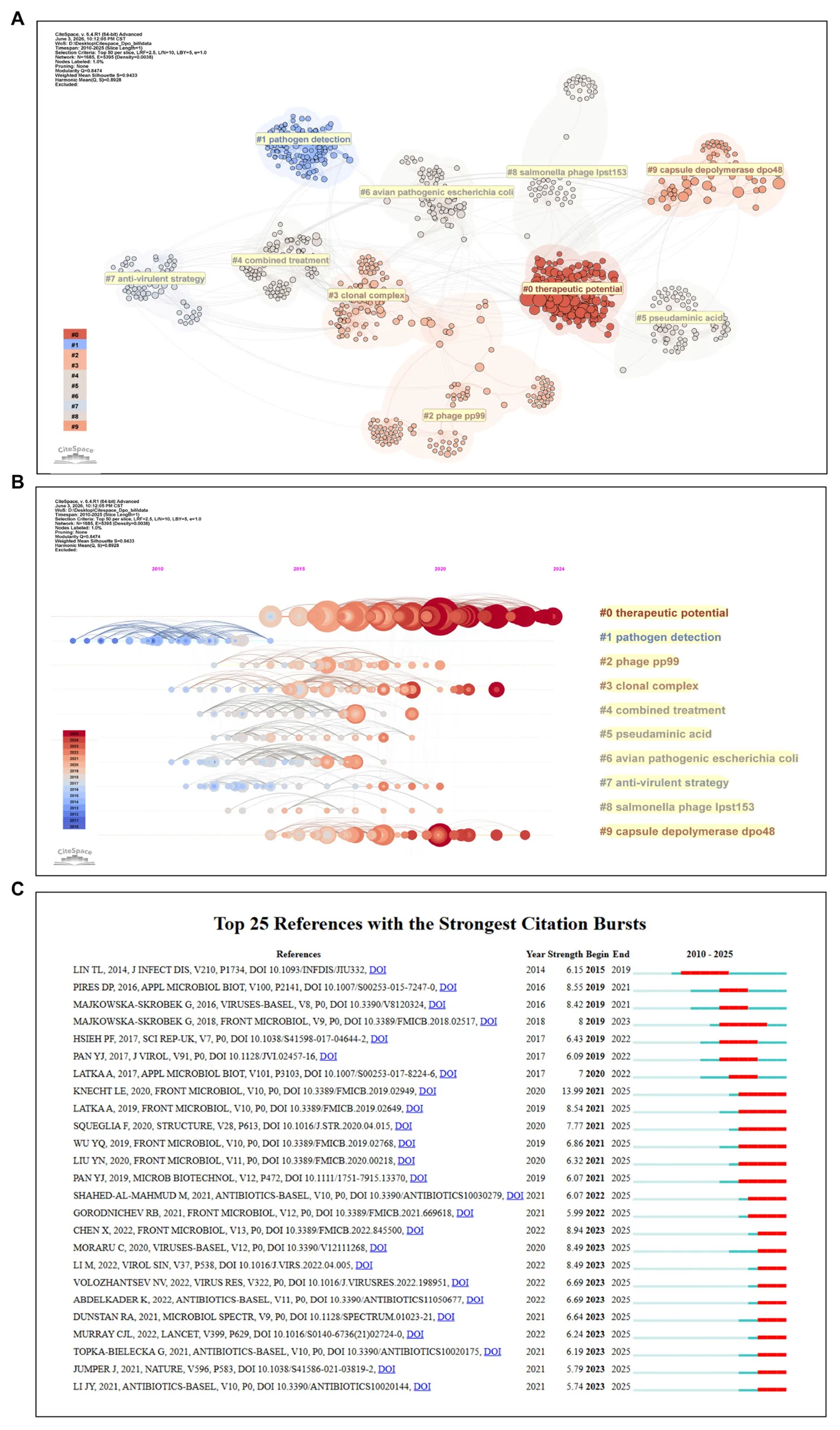
Reference co-citation network, temporal evolution, and citation burst analysis in phage depolymerase-related anti-infective research. **(A)** Visualization cluster map of co-cited references. Each circle represents a reference, and circles with the same color represent a cluster with the same topic. **(B)** Timeline view of reference co-citation analysis generated by CiteSpace. The horizontal axis represents publication year, and each horizontal line corresponds to a specific cluster. The position of each node indicates the time point at which the reference first appeared, node size represents citation frequency, and links indicate co-citation relationships. The color gradient from blue to red reflects the temporal evolution from earlier to more recent references. **(C)** Top 25 references with the strongest citation bursts. The strength value represents the intensity of citation bursts, and the red bars indicate the duration of the burst period.

### 3.7 Keyword co-occurrence and bacterial hotspot analysis

Keyword co-occurrence analysis identified 83 keywords, which were grouped into three major thematic clusters (Figure 8A). The red cluster, representing the foundational stage of depolymerase research, covers phage isolation, genomic characterization, and virological analysis (e.g., genome, host range, sequence analysis). Since many depolymerases are located within tail fiber or tail spike proteins, genome-based prediction and annotation have become critical starting points for identifying candidate enzymes (Han et al., 2026; Lawal and Goodridge, 2024; Hernandez-Morales et al., 2018; Hsieh et al., 2017). The green cluster, centered on phage therapy, biofilm control, and antibacterial applications, reflects the field's progression from descriptive phage characterization toward translational development and detailed depolymerase functional study. The blue cluster, focusing on bacteriophage-bacteria interactions, enzymatic function, and capsule degradation (e.g., glycosidase, protein structure), highlights growing interest in mechanistic and structural characterization. Collectively, these three clusters capture the major themes of phage depolymerase-related anti-infective research. Temporal distribution of high-frequency keywords (Figure 8B) shows that core terms such as bacteriophage, biofilm, and antibiotic resistance appeared early and persisted over time, whereas application-driven terms like capsule, virulence, and therapy have emerged more recently (post-2014), reflecting increasing interest in the anti-virulence activity and therapeutic potential of depolymerases.

**Figure 8:**
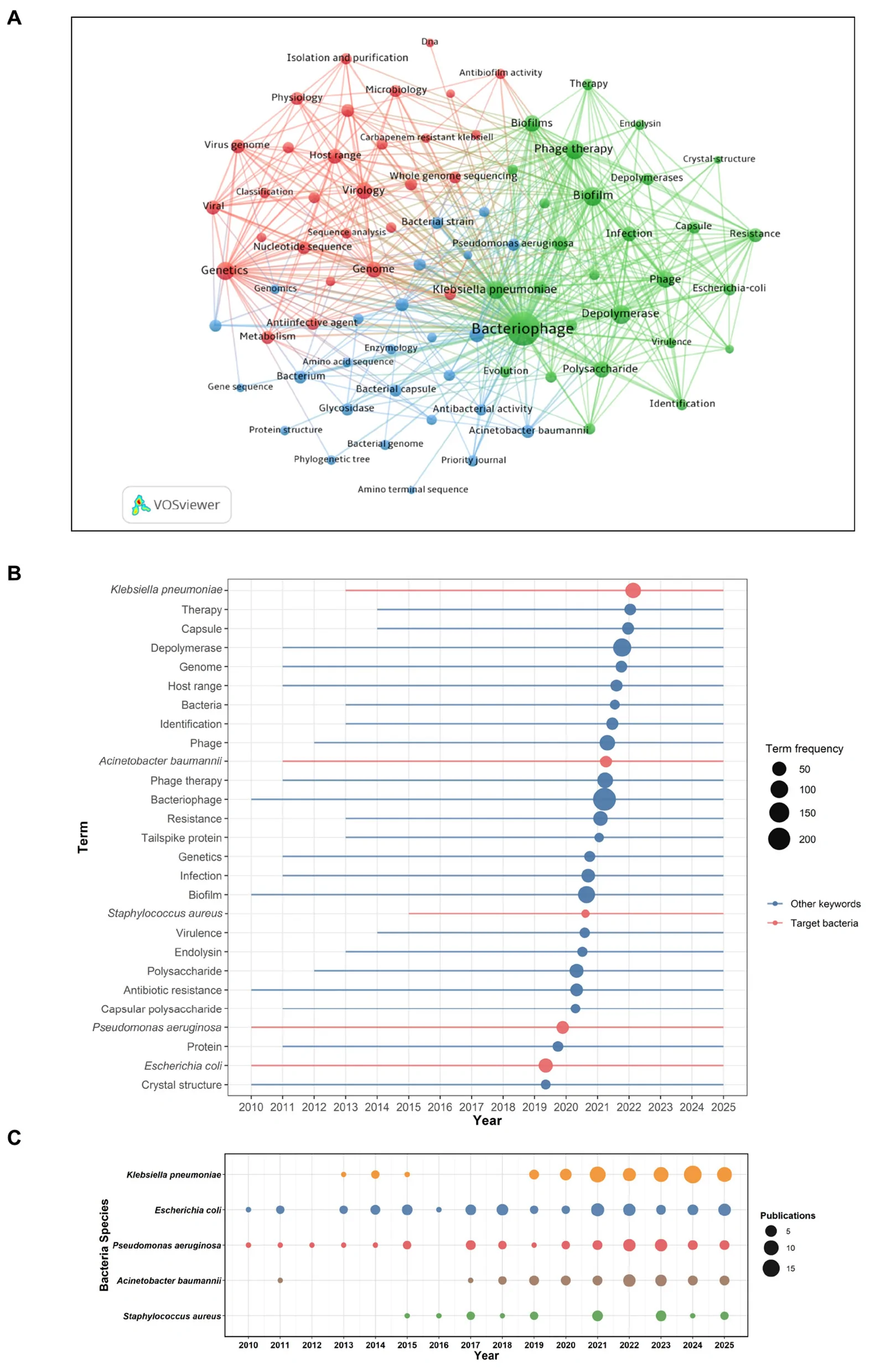
Keyword co-occurrence network and temporal evolution of research topics in phage depolymerase-related anti-infective research. **(A)** Keyword co-occurrence network generated by VOSviewer with a total link strength (TLS) > 220. Node size represents the frequency of keywords, links indicate co-occurrence relationships, and different colors represent distinct research clusters. **(B)** Trend topics in phage depolymerase-related anti-infective research. The x-axis represents publication year, and the y-axis represents keywords. Bubble size corresponds to keyword frequency. Red dots indicate target bacteria-related keywords, while blue dots represent other research-related keywords. **(C)** Annual publication trends of phage depolymerase-related studies targeting different bacterial species. Bubble size indicates the number of publications in a given year, and the numbers on the right represent the cumulative publication counts from 2010 to 2025.

Of note, terms such as phage therapy and endolysin show high-frequency co-occurrence with depolymerase-related keywords, indicating that research on depolymerases has expanded beyond the characterization of their intrinsic properties. Phage therapy showed 70 occurrences (rank 4, Figures 8A, B). In phage therapy research, depolymerases are a class of phage receptor-binding proteins (RBPs) that have been studied as determinants of phage host range and can be engineered to adjust or alter phage host range (Yehl et al., 2019; Łątka et al., 2021; Dams et al., 2019). Endolysin, another major class of phage-derived antibacterial enzymes, also appeared prominently (21 occurrences, rank 22, Figures 8A, B). Although endolysins degrade cell wall peptidoglycan rather than the bacterial surface and extracellular polysaccharides that serve as depolymerase substrates, their frequent co-occurrence with depolymerase-related terms indicates substantial thematic overlap in the literature, particularly in the context of biofilm eradication and combination therapeutic strategies (Olsen et al., 2018; Husain et al., 2025; Danis-Włodarczyk et al., 2021; Zhang et al., 2025c).

A subset of keywords corresponded to bacterial species (Figures 8B, C), reflecting the target organisms of the included studies. *Klebsiella pneumoniae* (*K. pneumoniae*) led with 67 occurrences, followed by *Escherichia coli* (*E. coli*, 52), *Pseudomonas aeruginosa* (*P. aeruginosa*, 36), *Acinetobacter baumannii* (*A. baumannii*, 30) and *Staphylococcus aureus* (*S. aureus*, 18). Temporally, studies on *E. coli* and *P. aeruginosa* appeared relatively early and remained continuous throughout the study period, whereas *K. pneumoniae* and *A. baumannii* exhibited a marked increase in attention after 2018. In particular, *K. pneumoniae* became increasingly prominent in recent years, reflecting a growing research focus on clinically important encapsulated and biofilm-forming pathogens (Squeglia et al., 2020; Lee et al., 2017).

Overall, the keyword analysis revealed that phage depolymerase-related anti-infective research is primarily centered on three thematic pillars: phage genomic characterization, antibacterial and antibiofilm applications, and mechanistic studies of depolymerase activity–with increasing attention toward anti-infective applications against clinically important encapsulated pathogens.

## 4 Discussion

### 4.1 Research growth and major contributors

This study retrieved publications on phage-derived depolymerases in anti-infective therapy from 2010 to 2025, integrating data from WOSCC and Scopus to enhance the comprehensiveness of the dataset. The analysis systematically examined publication trends, major contributors (countries, institutions, authors, and journals), co-citation and citation burst patterns, and keyword co-occurrence and bacterial species hotspots. The analytical corpus comprises 326 publications–a modest figure relative to the 6538 publications in phage overall (2001–2021) (Maimaiti et al., 2023). Yet it is comparable in scale to other phage-related subfields over similar periods, such as the phage-gut microbiota interactions (687 publications) (Kuang et al., 2026) and phage applications against *A. baumannii* (264 publications) (Jiang et al., 2025), confirming depolymerase research as a distinct and specialized niche. These recent emergence of analogous bibliometric analysis on phage subfields signals the proliferation of the overall phage field into specialized fronts. Our work, arising from the same context, is distinct in focusing on the phage-encoded depolymerase itself–a molecular-level perspective that complements existing application-oriented analyses. Meanwhile, the exponential growth of this field, conforming to Price's law (Figure 2), indicates a clear transition toward accelerated expansion.

The temporal distribution of publications indicates that research activity remained relatively limited before 2016, followed by steady growth and a marked acceleration after 2020 (Figure 2). The inflection point may be attributed to several converging factors. The global spread of MDR and rising antimicrobial resistance have intensified the demand for novel antibacterial strategies, crystallized by the WHO's 2017 priority list of critical-priority pathogens, including carbapenem-resistant *A. baumannii, P. aeruginosa*, and Enterobacteriaceae (Tacconelli et al., 2018). The COVID-19 pandemic further heightened clinical urgency, as immunosuppressed patients faced elevated risks of secondary bacterial infections, coupled with limited effective antibiotics, driving need in therapeutic alternatives (Nazir et al., 2023; Crook et al., 2023). Against this backdrop, phage-derived depolymerases have attracted growing attention as promising anti-infective agents, with their anti-virulence efficacy demonstrated in animal infection models, particularly under immunosuppressive conditions (Pan et al., 2015; Lin et al., 2014; Wang et al., 2024a; Tacconelli et al., 2018).

The major contributors to this field, spanning countries/regions, institutions, authors, and journals, are detailed in Tables 1–3, and 5. At the country/region level, China and the USA were the major drivers of publication output, while European countries such as Belgium and Poland demonstrated substantially higher average citation impact. Although these countries/regions were all main contributors and important nodes in the collaboration network (Figure 3D), these findings indicate that research impact and productivity were not fully equivalent. This discrepancy is consistent with the observation by Fox et al., concerning the arbitrariness of bibliometric parameters, in that reliance on any single indicator may yield an incomplete assessment of research contributions (Fox and Seifert, 2024). As phage-derived depolymerase research is closely connected to the broader field of phage therapy–a connection supported by the co-occurrence shown in Figures 8A, B–its development is shaped not only by scientific motivation but also by national clinical traditions and regulatory policies. The strong citation performance of Polish and Belgian groups reflect their early foundations in phage therapy, including the Wrocław phage therapy center in Poland, which has operated since the 1950s (Zaczek et al., 2022a,b), and Belgium's Queen Astrid Military Hospital (QAMH), which was among the first hospitals to reintroduce clinical phage therapy under the “unproven interventions” framework in 2007 (Djebara et al., 2019). The USA's sustained leadership in phage therapy clinical trials, as demonstrated by bibliometric analysis, has similarly provided a favorable basis for continued productivity in this derivative depolymerase field (Dagli et al., 2024; Maimaiti et al., 2023).

By comparison, although China established the Shanghai Institute of Phage and Drug Resistance in 2017 and has since expanded phage-related research, its national regulatory framework (State Council Decree No. 818) for phage therapy only began to take shape in 2026 (Jixin et al., 2026), whereas Belgium had already established the “Magistral Phage” framework in 2018 to enable the magistral preparation of personalized phage medicines (Pirnay et al., 2018; Verbeken and Pirnay, 2022). This later regulatory development likely contributed to a delayed surge in China's depolymerase-related publications, which only began to increase substantially after 2021 (Figure 3B). Given their short citation window–citation peaks typically occur 3-5 years after publication and a minimum 3-year window is recommended for impact assessment–these recent publications explain China's observed mismatch between publication volume and citation accumulation (Supplementary Figure S2) (Wang, 2013). Moreover, China had relatively fewer internationally co-authored publications (Figure 3A); as internationally co-authored papers generally receive significantly more citations, this collaboration pattern provides an additional possible explanation for China's relatively lower per-publication citation performance (Wang et al., 2024b; Islam et al., 2024). These findings highlight the potential value of strengthening cross-border partnerships to enhance the visibility and impact of research in this domain.

At the institutional and author levels, research activity was concentrated within several core teams, particularly the Polish University of Wrocław group and the Russian Academy of Sciences group (Table 2). Among these, Drulis-Kawa Zuzanna from the University of Wrocław ranked first in volume, citations, and H-index, whereas her colleagues Maciejewska Barbara and Łątka Agnieszka achieved remarkably high CPP (Table 3), illustrating distinct impact archetypes that underscore the need for multidimensional evaluation, combining size-dependent (e.g. H-index) and size-independent (e.g. CPP) indicators (Fox and Seifert, 2024; Katz, 2006; Brito and Rodríguez-Navarro, 2021). However, research activity in other productive countries/regions was more dispersed, with no single dominant center emerging, consistent with the absence of their institutions and authors from the top rankings (Tables 1–3). Overall, this author productivity distribution follows Lotka's law, whereby a minority of core authors drives the majority of output in a given field (Vlachý, 1978; Kumar et al., 1998). In the context of phage depolymerase research, this pattern reflects the resource-intensive nature of experimental work, wherein sustained productivity is contingent on long-term funding, specialized facilities, and established collaborative networks that disproportionately favor already-productive groups (Wuchty et al., 2007; Newman, 2001). Therefore, keeping abreast of the scientific output of these core teams is an efficient strategy for following cutting-edge advances, and collaborating with them has the potential to promote research progress and enhance scholarly impact (Chen et al., 2025; Zhou et al., 2025).

The journal landscape is dominated by specialized microbiology and virology journals, with *Frontiers in Microbiology, Viruses-Basel*, and *Antibiotics-Basel* serving as the primary publication venues (Table 5 and Figure 6A). The Bradford distribution observed here (Table 4)–with 7 core journals producing 33.7% of all publications–confirms the characteristic concentration of literature in a small number of core journals, a pattern consistent with findings in other medical specialties (e.g., pharmacy and neurosurgery) (Rao, 2006; Adeosun et al., 2026; Venable et al., 2016). Continuous attention to these core journals may help researchers remain informed about emerging developments in the field.

Taken together, these bibliometric characteristics provide a foundational overview of the global research landscape, highlighting both the concentrated nature of research activity and the growing momentum in this domain.

### 4.2 Research hotspots and frontiers

In bibliometric analysis, co-cited references and keyword co-occurrence provide valuable perspectives on major research trends and focal areas, which are essential for a comprehensive understanding of a field's evolution. High-frequency keyword and co-cited reference analysis can help identify major themes, and the temporal keyword and reference analysis demonstrated an evolution of research priorities within a field. The research hotspots in phage depolymerase-related anti-infective research can be organized into three major thematic directions, yet a critical reading of these clusters reveals both notable achievements and significant gaps (Figure 8A).

The first theme (red cluster)–phage discovery and genomic characterization–represents the foundational and most mature stage (Figure 8A). The dominance of this cluster reflects that depolymerase discovery remains primarily driven by culture-dependent phage isolation and genome sequencing. As phages are the most abundant biological entities (~10^31^ particles), only a tiny fraction of natural phage diversity and its associated depolymerase repertoire has been characterized and sequencing new isolates will continue to uncover novel genes and depolymerases (Hatfull, 2015; Ngiam et al., 2022). Most citation burst references involve the identification and characterization of depolymerases, indicating that the search for depolymerases with low sequence homology, distinct substrate specificities, or novel structural features continues to drive the field (Figure 7C).

The second theme (green cluster)–antibacterial and antibiofilm applications–shows that the field has progressed from descriptive phage characterization toward anti-infective research, especially against multidrug-resistant bacteria (Figure 8A). This anti-infective research interest stems from the biological role of depolymerases during phage infection of bacteria. In the initial infection step, RBPs, including TFPs and TSPs, specifically recognize bacterial surface and extracellular polysaccharides (CPS, EPS, and LPS O-antigens) (Peters et al., 2024; Boeckaerts et al., 2021; Pas et al., 2023; Matusiak et al., 2025; Ostenfeld et al., 2024; Yehl et al., 2019; Lai et al., 2016). This specific intermolecular interaction determines the corresponding specificity between depolymerases (and their phages) and bacterial species with specific serotypes. Many RBPs possess polysaccharide depolymerase domains that degrade these barriers, thereby increasing bacterial susceptibility to antibiotics and host immune clearance (Liu et al., 2019a,b; Borzilov et al., 2025; Abdelkader et al., 2022). Thus, depolymerases not only function as host-recognition determinants in phage therapy but also hold promise as independent anti-virulence agents (Campos et al., 2004; Lee et al., 2013). On the one hand, RBPs are often engineered to alter or expand phage host range in phage therapy (Yehl et al., 2019; Łątka et al., 2021; Dams et al., 2019). On the other hand, the host-specific recognition of depolymerases was initially explored for pathogen detection (Singh et al., 2012; Javed et al., 2013; Kunstmann et al., 2018), and later their anti-virulence potential drew attention (Figure 8B) (Han et al., 2026; Kunisch et al., 2024). Together, despite their promising potential, both therapeutic avenues–phage host-range engineering and depolymerase-based anti-virulence strategies–are still confined to laboratory investigations, and considerable efforts are needed to bridge the gap toward clinical translation.

The third theme (blue cluster)–mechanistic and structural characterization–represents the most rapidly advancing frontier (Figure 8A). Structural studies show that depolymerases typically form a homotrimer, with the N-terminal domain (NTD) serving as an anchor attaching to the phage particle, the central receptor-binding domain (RBD) possessing catalytic activity, and the C-terminal domain (CTD) responsible for trimerization and substrate recognition (Seul et al., 2014; Squeglia et al., 2020; Lee et al., 2017); moreover, the RBD holds a characteristic right-handed parallel β-helix fold, while the CTD often contains carbohydrate-binding module (CBM) and/or lectin-like modules (Otwinowska et al., 2026; Squeglia et al., 2020). Collectively, these structural features provide important clues to the mechanisms of substrate recognition and polysaccharide cleavage in several depolymerases (Pas et al., 2023; Lai et al., 2016; Lee et al., 2017; Otwinowska et al., 2026). Notably, recent studies have revealed that the CTD may influence the range of capsular polysaccharide specificity, and an isolated CTD fragment alone has also been found to possess enzymatic activity, suggesting that the CTD plays a more active and diverse role than previously appreciated (Kravchuk et al., 2026; Otwinowska et al., 2026). These structural insights have been applied to phage therapy–a high-frequency keyword in the co-occurrence analysis (Figures 8A, B)–where modular RBP engineering has been used to switch capsular serotype specificity, enabling well-characterized phages to be repurposed against pathogens outside their original host range (Yehl et al., 2019). For example, the N-terminal anchor of K11gp17 (the RBP of phage K11, which lyses K11-type *K. pneumoniae*) was retained, while its enzymatic domain (RBD and CTD) was replaced with that of KP32gp37 (the RBP of phage KP32, which targets K21-type *K. pneumoniae*). The resulting chimeric phage K11 lysed K3-type but not K11-type *K. pneumoniae* (Łątka et al., 2021). Given the numerous clinically important pathogens and their diverse serotypes, along with the even more diverse array of corresponding depolymerases, the experimentally identified serotype-specific depolymerases and their structural knowledge represent only a minute fraction. In addition, CPS/EPS-related terms, such as capsule and biofilm, appear more frequently in keyword analyses and have received greater research attention, whereas LPS O-antigen-related studies are comparatively limited (Figures 8A, B) (Zhang et al., 2026; Knecht et al., 2020). Thus, more extensive studies are needed to characterize the unknown and understudied types of depolymerases (Cheetham et al., 2024; Lam et al., 2022).

Machine-learning-related keywords such as machine learning and deep learning appeared only a few times in the analyzed literature (Supplementary Table S1), and only in publications from the past one to two years. This pattern is consistent with the recent application of machine learning in this field. While its bibliometric visibility remains limited, machine learning is nonetheless a cutting-edge and important development in this field. As wet-lab experiments are time-consuming, several machine learning-based prediction tools for depolymerases and their substrate specificity (e.g., DePP, DepoScope, PhageDPO, and DposFinder) have been developed to accelerate discovery; however, their training datasets are heavily Klebsiella-biased, and most in silico predictions lack experimental validation (Magill and Skvortsov, 2023; Concha-Eloko et al., 2024; Vieira et al., 2025; Shen et al., 2026). A recent large-scale experimental effort, DepoCatalog, has begun to address this gap by identifying 129 *Klebsiella* phage depolymerases (72 novel and 57 literature-derived); of these, 90 (72 novel ones plus 18 literature-derived after removing highly homologous sequences) were recombinantly produced and experimentally validated for their capsule specificity, expanding the validated coverage to 75 K-locus (KL) capsule types (Otwinowska et al., 2026). Although this still represents less than half of the reported capsular loci–at least 161 distinct KL have been defined to date based on the latest Kaptive database (v3.2.0)–such experimentally validated datasets are indispensable for refining computational models and moving toward more reliable, generalizable predictions (Lam et al., 2022; Otwinowska et al., 2026).

The prominent co-occurrence of endolysin in the keyword network invites a comparative perspective between these two classes of phage-derived enzymes (Figures 8A, B). Endolysins hydrolyze cell wall peptidoglycan and directly lyse Gram-positive bacteria, whereas depolymerases degrade surface and extracellular polysaccharides without direct bactericidal activity (Qian and Zheng, 2026; Zhang et al., 2025c). Their enzymatic activities were first observed around the similar time (endolysins: 1957; depolymerases: 1956) (Ralston et al., 1957a,b; Adams and Park, 1956), yet *in vivo* efficacy was demonstrated with a 13-year gap (endolysins: 2001; depolymerases: 2014) (Nelson et al., 2001; Lin et al., 2014; Bansal et al., 2014). Consequently, endolysins have advanced into clinical trials (e.g., CF-301, CF-296 and SAL200) (Jun et al., 2017; Fowler et al., 2024; Indiani et al., 2019), while depolymerases remain preclinical, a disparity likely attributable to their bactericidal versus anti-virulence mechanisms. However, the clinical applicability of endolysins is largely confined to Gram-positive bacteria, as the outer membrane of Gram-negative pathogens substantially impedes exogenous endolysin access to the peptidoglycan layer (Schmelcher et al., 2012; Liu et al., 2023). Beyond this comparison, the endolysin-depolymerase co-occurrence literature in our dataset not only highlights the antimicrobial potential of both enzymes but also reveals their synergistic effects in biofilm eradication, pointing to another combination strategy for depolymerases beyond their well-recognized partnership with antibiotics, such as the endolysin LysK-depolymerase DA7 and endolysin LysPB32-depolymerase DpolP22 combinations (Olsen et al., 2018; Kim et al., 2023; Zhang et al., 2025c; Husain et al., 2025; Danis-Włodarczyk et al., 2021).

Bacterial hotspot analysis provided additional insight into the major target pathogens investigated in polysaccharide depolymerase research (Figure 8C). *K. pneumoniae, E. coli, P. aeruginosa, A. baumannii*, and *S. aureus* are the most frequently studied pathogens. *K. pneumoniae* is the most prominent, particularly in recent years, which is biologically plausible given its heavy encapsulation and the pivotal role of capsule in virulence and immune evasion; capsule-targeting depolymerases are therefore particularly promising against this pathogen (Zhang et al., 2025c; Maciejewska et al., 2023; Pyra et al., 2017; Wu et al., 2023; Costa et al., 2022; Brzozowska et al., 2017). Attention to *A. baumannii* has risen increasingly after 2017, mirroring the urgent need for novel strategies against MDR nosocomial pathogens as prioritized by the WHO (Tacconelli et al., 2018; Shokrollahi and Singleton, 2017). *E. coli* and *P. aeruginosa* have been consistently represented, reflecting their long-standing importance as both model organisms and biofilm-forming pathogens (Park and Park, 2021; Witte et al., 2021; Huan et al., 2022; Zhang et al., 2023; Lin et al., 2025; Sillankorva et al., 2022). Overall, the bacterial hotspot of depolymerases research is clearly concentrated on Gram-negative bacteria (Figure 8C and Supplementary Table S1), consistent with previous literature (Wang et al., 2024a; Magill and Skvortsov, 2023). Two non-mutually exclusive explanations may account for this gap. First, the distinct cell envelope architecture of Gram-positive bacteria–particularly their thicker peptidoglycan layer–may affect the evolutionary frequency of depolymerases in phages infecting Gram-positive bacteria, although EPS and CPS occur in both Gram-positive and Gram-negative bacteria (Nwodo et al., 2012). Second, as noted above, the greater efficacy of endolysins in Gram-positive bacteria may have diverted research attention away from depolymerases for these pathogens (Sabur et al., 2025).

In summary, the three thematic clusters form a coherent knowledge pipeline. Phage discovery (red cluster) provides the primary source of polysaccharide depolymerases, which directly supports application-oriented studies (green cluster) that test their antibacterial and antibiofilm potential. The limitations encountered in these applied studies, however, highlight the need for mechanistic and structural characterization (blue cluster) to guide further optimization. Together, the knowledge generated by these three clusters constitutes the core content of a conceptual bacteria-phage-depolymerase-application matching database. While existing resources such as Kaptive provide genotypic information based on bacterial surface polysaccharide loci (Lam et al., 2022) and DepoCatalog offers experimentally validated collections of *K. pneumoniae* phage depolymerases (Otwinowska et al., 2026), we propose a conceptual framework that systematically links capsular genotype, enzyme substrate specificity, and therapeutic application contexts–an integrative matching logic not currently provided by either resource (Figure 9). This framework is a conceptual, forward-looking synthesis derived from the three keyword co-occurrence clusters (Figures 8A, B) and the co-citation and citation-burst analyses (Figures 7A, B), combined with the latest literature, rather than a direct quantitative output of the bibliometric software. Nevertheless, we illustrate it here with a representative example that traces from a K2-type *K. pneumoniae* host (strain K7) and its lytic phage GH-K3 to the depolymerase Depo32 and its validated *in vivo* protection, with the relevant literature (Figure 9) (Cai et al., 2019, 2023). Expanding this framework to cover more serotypes and clinically relevant strains, while further integrating enzyme activity, mechanism, and structural features, would strengthen its utility in guiding depolymerase-based anti-infective strategies.

**Figure 9:**
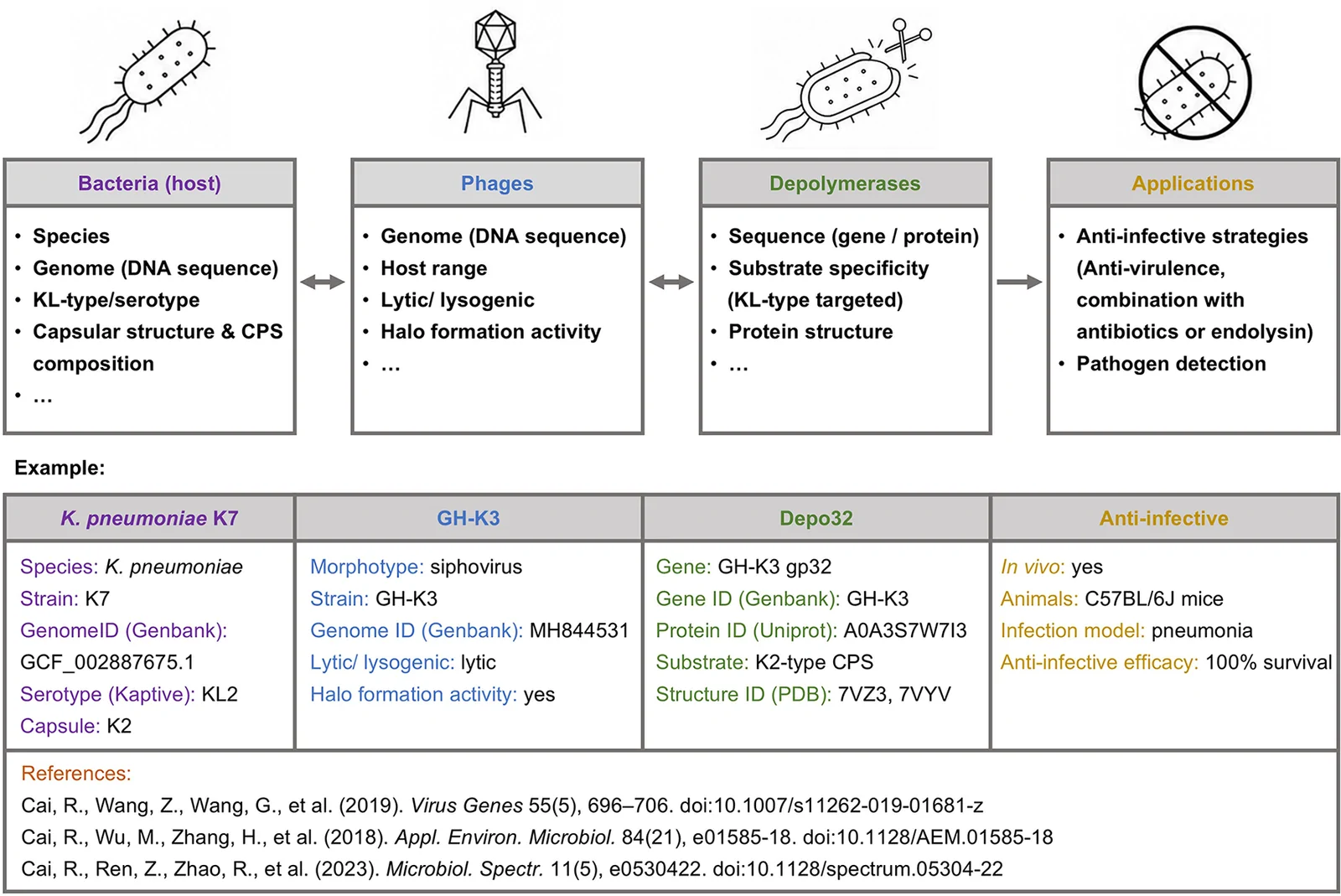
Conceptual framework for a bacteria-phage-depolymerase-application matching database, illustrating the essential data layers for bacterial hosts, phages, and depolymerases, their interconnections, and downstream applications. The lower panel provides a representative example, illustrating how the abstract data layers correspond to actual database fields and application contexts.

### 4.3 Challenges

The under-representation of certain terms in the keyword analysis of the analyzed literature on depolymerase-based anti-infective research (Supplementary Table S1) reveals a clear gap in preclinical and translational aspects, with safety appearing fewer than 10 times, formulation only twice (both in review articles), and manufacturing absent. This paucity suggests that preclinical and translational aspects remain understudied and that the field is still in its early stages, despite rapid growth in related publications. No depolymerase has yet obtained Investigational New Drug (IND) clearance or entered clinical trials. Accordingly, the challenges outlined below, presented as complementary narrative and ranging from biochemical limitations to preclinical hurdles, represent critical steps toward clinical translation.

(1) Specificity. The substrate specificity of naturally occurring depolymerases–while advantageous for microbiome preservation–results in inherently narrow target spectra (Zhang et al., 2025a; Leiman et al., 2007; Hsieh et al., 2017; Lai et al., 2016; Borzilov et al., 2025), limiting their efficacy against the highly heterogeneous surface polysaccharides displayed by the numerous clinical serotypes of a given pathogen. Analogous to phage therapy, a cocktail design employing multiple polysaccharide depolymerases could simultaneously target bacteria of various capsular types (Wang et al., 2024a; Guo et al., 2023). Furthermore, although extended substrate specificity of depolymerase has been reported, this cross-reactivity between bacterial species is attributable to similar capsule structures (Maciejewska et al., 2023). Based on a thorough understanding of the structure-activity relationships of depolymerases, protein engineering approaches–including domain swapping, directed evolution, and computational design–hold promise for modulating the substrate specificity of polysaccharide depolymerases (Maciejewska et al., 2023; Ye et al., 2024).

(2) Formulation. Free depolymerases, detached from the stabilizing structural context of the phage virion, may exhibit reduced structural stability, potentially compromising their storage stability and functional persistence in the complex biochemical environment of infection sites (Maciejewska et al., 2023; Liu et al., 2025). Current therapeutic practice in animal models predominantly relies on liquid-based formulations for parenteral injection, with limited exploration of non-invasive oral or topical delivery routes (Wang et al., 2024a). Several studies have investigated the stability of depolymerases under various conditions–including long-term storage, pH, temperature, buffer composition, and the presence of metal ions, surfactants, reductants, and protease inhibitors (Wang et al., 2024a; Chen et al., 2022; Majkowska-Skrobek et al., 2018; Waseh et al., 2010; Kassa and Chhibber, 2012)–yet comprehensive formulation design and systematic evaluation remain lacking. Compared with the well-established formulation science for therapeutic monoclonal antibodies and other protein biologics, the depolymerase field still exhibits a considerable gap (Martin et al., 2023; Majkowska-Skrobek et al., 2018).

(3) Safety. As exogenous proteins, phage depolymerases carry inherent immunogenicity risks, and their overall safety profiles have not yet been systematically evaluated (Maciejewska et al., 2018; Bansal et al., 2014; Plat et al., 2026; Volozhantsev et al., 2025). Current evidence from animal studies at therapeutic doses suggests promising preliminary safety profiles, with no significant differences in blood biochemical markers, histopathological damage, survival rate, body weight, or abnormal behavior observed between treated and control groups (Bansal et al., 2014; Shahed-Al-Mahmud et al., 2021; Pan et al., 2015; Liu et al., 2019a; Lin et al., 2017). Nevertheless, the safety risks associated with higher doses or repeated administration remain to be assessed in larger-scale experiments.

(4) Manufacturing. Recombinant expression remains the primary approach for obtaining large quantities of polysaccharide depolymerases (Olszak et al., 2017; Lin et al., 2017; Hsieh et al., 2017; Greenfield et al., 2019). However, inadequate purification or contamination during downstream processing could increase immunogenicity and allergenicity, necessitating rigorous procedures and multiple separation, filtration, and sterilization steps in compliance with Good Manufacturing Practices (GMPs) (Klimyuk et al., 2014). Moreover, challenges related to large-scale production, purification scalability, process optimization, and quality control remain to be further explored to facilitate clinical translation.

Addressing these hurdles is essential to advance depolymerases toward clinical application against drug-resistant bacteria. Given several endolysins–another class of phage-encoded antibacterial enzymes–have already entered clinical trials (Qian and Zheng, 2026), their development experience offers valuable lessons for depolymerases. For instance, SAL200, an endolysin drug that has advanced into clinical trials, has its active ingredient SAL-1 formulated into a stable injectable pre-formulation uniquely containing calcium ions as an activity enhancer and Poloxamer 188 as a stabilizer (Jun et al., 2013). Subsequently, SAL200 underwent systematic Good Laboratory Practice (GLP)-compliant preclinical safety evaluation in rodents and dogs, showing no significant toxicity except transient, reversible signs upon repeated dosing (Jun et al., 2014). Pharmacokinetic studies in monkeys further established dose-proportional exposure and tolerability up to 80 mg/kg (Jun et al., 2016). Following these preclinical studies, SAL200 has completed Phase 1 (single-dose, ClinicalTrials.gov identifier NCT01855048, and multiple-dose, NCT03446053) and 2a clinical trials (NCT03089697) (Jun et al., 2017). This stepwise progression–from rational formulation design and preclinical characterization through clinical evaluation–provides a concrete roadmap for depolymerase development.

## 5 Novelty statement

Several narrative reviews have summarized the progress and challenges of depolymerase research (Guo et al., 2023; Islam et al., 2024; Oliveira et al., 2022; Topka-Bielecka et al., 2021); however, their conclusions rely largely on expert interpretation and selected literature. This systematic review, based on bibliometric analysis, integrates data-driven results with the latest literature to provide new insights and explain the underlying reasons. Its main contributions are as follows.

First, this study quantifies the total publication volume and temporal trajectory of this field and, using Price's law, demonstrates the field's quantitative growth and the recent increase in research activity.

Second, this study uses temporal changes in national/regional output to reveal distinct developmental trajectories across countries/regions. Importantly, building on the co-occurrence of depolymerase and phage therapy research, we link depolymerase research with phage therapy research, it further explains the reasons for these differences across countries/regions–an aspect that has not been previously analyzed.

Third, identifying core institutions and authors helps recognize research teams that contribute substantially to the research frontiers of this field. Beyond narrative reviews, which summarize specific findings, tracking the outputs of these core teams can help researchers follow high-quality frontier advances and identify promising research questions or directions in this field.

Fourth, although the anti-*K. pneumoniae* activity of depolymerases has been emphasized (Zhang et al., 2025c; Otwinowska et al., 2026), this study uses keyword data to quantify research attention and potential across different bacterial species, and tentatively suggests reasons for the uneven bacterial distribution and the marked underrepresentation of Gram-positive bacteria.

Fifth, the high-frequency co-occurrence of endolysin highlights the value of combining endolysins with depolymerases, despite limited reports on their combination (Olsen et al., 2018; Kim et al., 2023). Although previous narrative reviews have also mentioned or discussed the two together (Zhang et al., 2025c; Husain et al., 2025; Danis-Włodarczyk et al., 2021), this study emphasizes that the more advanced clinical development of endolysins can serve as a reference for the translational development of depolymerases.

Sixth, through keyword clustering and temporal analysis, this study quantitatively delineates the evolution of research themes and proposes a conceptual framework for an integrative bacteria–phage–depolymerase–application matching database, which is distinct from Kaptive and DepoCatalog (Lam et al., 2022; Otwinowska et al., 2026).

Collectively, these contributions provide temporally resolved, network-based, and data-driven evidence that extends beyond previous qualitative syntheses, while the complementary narrative included in the Discussion of this systematic review further enhances the interpretation and contextualization of the findings.

## 6 Limitations

This bibliometric analysis provides a systematic, multi-dimensional overview of global research on phage-encoded polysaccharide depolymerases as anti-infective agents, integrating publication trends, collaboration networks, thematic evolution, and emerging research frontiers over a 16-year period. Nevertheless, several methodological considerations should be noted when interpreting these findings. First, although the literature search encompassed two widely used and complementary bibliographic databases–WOSCC and Scopus–it may not have captured all relevant publications, particularly those published in languages other than English or indexed in region-specific databases; future updates could incorporate additional sources such as PubMed or regional citation indices to enhance coverage. Second, the search strategy, despite employing comprehensive Boolean operators, may have inadvertently omitted a small number of relevant publications due to the inherent trade-off between search sensitivity and specificity–a well-recognized challenge in systematic bibliometric investigations. Third, bibliometric indicators such as citation counts are influenced by publication recency and field-specific citation practices, indicating that these metrics should be interpreted as relative rather than absolute measures of research impact. Fourthly, the analysis of research hotspots and trends was based primarily on keyword co-occurrence and citation patterns, which capture structural and quantitative dimensions of the literature; integrating qualitative approaches such as critical reviews or expert elicitation in future work could provide complementary depth. Finally, as a descriptive and correlational method, bibliometric analysis cannot establish causation; the factors discussed here are therefore plausible hypotheses, not causal mechanisms. These limitations, while constraining certain aspects of the present analysis, also delineate productive avenues for subsequent bibliometric and qualitative investigations in this domain.

## 7 Conclusions

Using bibliometric methods, we integrated and analyzed the global corpus of English-language articles and reviews on phage depolymerase-related anti-infective research published from 2010 to 2025. The results delineate international collaboration patterns, productivity concentrations, journal ecosystems, and thematic evolution within this rapidly expanding field. Scientific output remains unevenly distributed, with China and the USA leading in publication volume, while European countries such as Belgium and Poland demonstrate higher citation impact. Three thematic pillars–genomic discovery of tail-associated depolymerases, antibacterial and antibiofilm applications, and mechanistic/structural characterization–define the contemporary knowledge structure, with clear evolutionary trends from basic discovery toward application-oriented research. While current research has made notable progress, substantial further work is still required. The structural determinants underlying the diverse substrate specificities of depolymerases remain to be clarified in greater detail to consolidate the knowledge base. Preclinical studies on formulation development, safety evaluation, and scale-up manufacturing should also be advanced, drawing on the development experience of endolysins, to facilitate the clinical translation of depolymerases.

## Data Availability

The original contributions presented in the study are included in the article/Supplementary material, further inquiries can be directed to the corresponding authors.
