# Supplementary material for: Global trends in phage-derived depolymerase research for anti-infective applications: a bibliometric analysis
Source: Front Microbiol. 2026 Oct 8;17:1947768. doi: 10.3389/fmicb.2026.1947768 (PMC13687390; doi:10.3389/fmicb.2026.1947768)
Supplement: Supplementary file 1 [file Data_Sheet_1.pdf]

Supplementary Material

1 SUPPLEMENTARY FIGURE

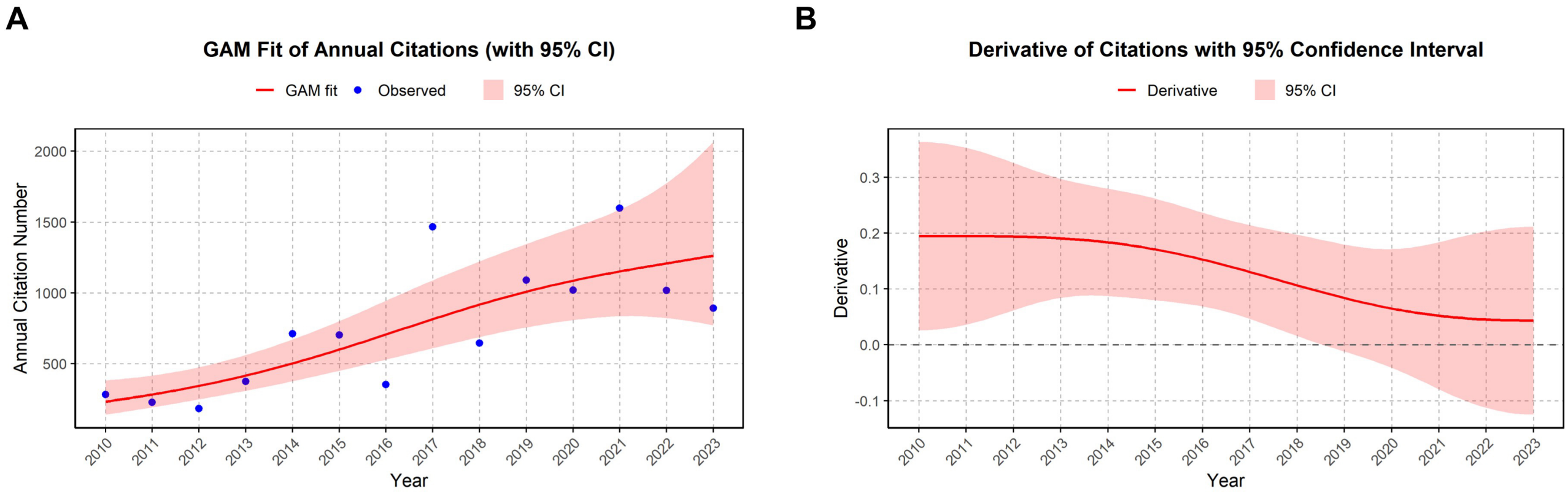

Figure S1. Annual citation trends and first-derivative analysis. **(A)** Observed annual citation counts (blue dots) and negative binomial GAM-fitted smooth curve (red line) with 95% confidence interval (red ribbon). The smooth term used $k = 5$. Years 2024–2025 were excluded because citation accrual is incomplete. **(B)** First derivative of the fitted GAM curve (red line) with 95% confidence interval (red ribbon). The dashed horizontal line indicates zero derivative. A statistically significant peak is defined as a point where the derivative changes from positive to negative and the 95% CI excludes zero on both sides. No statistically significant peaks were detected.

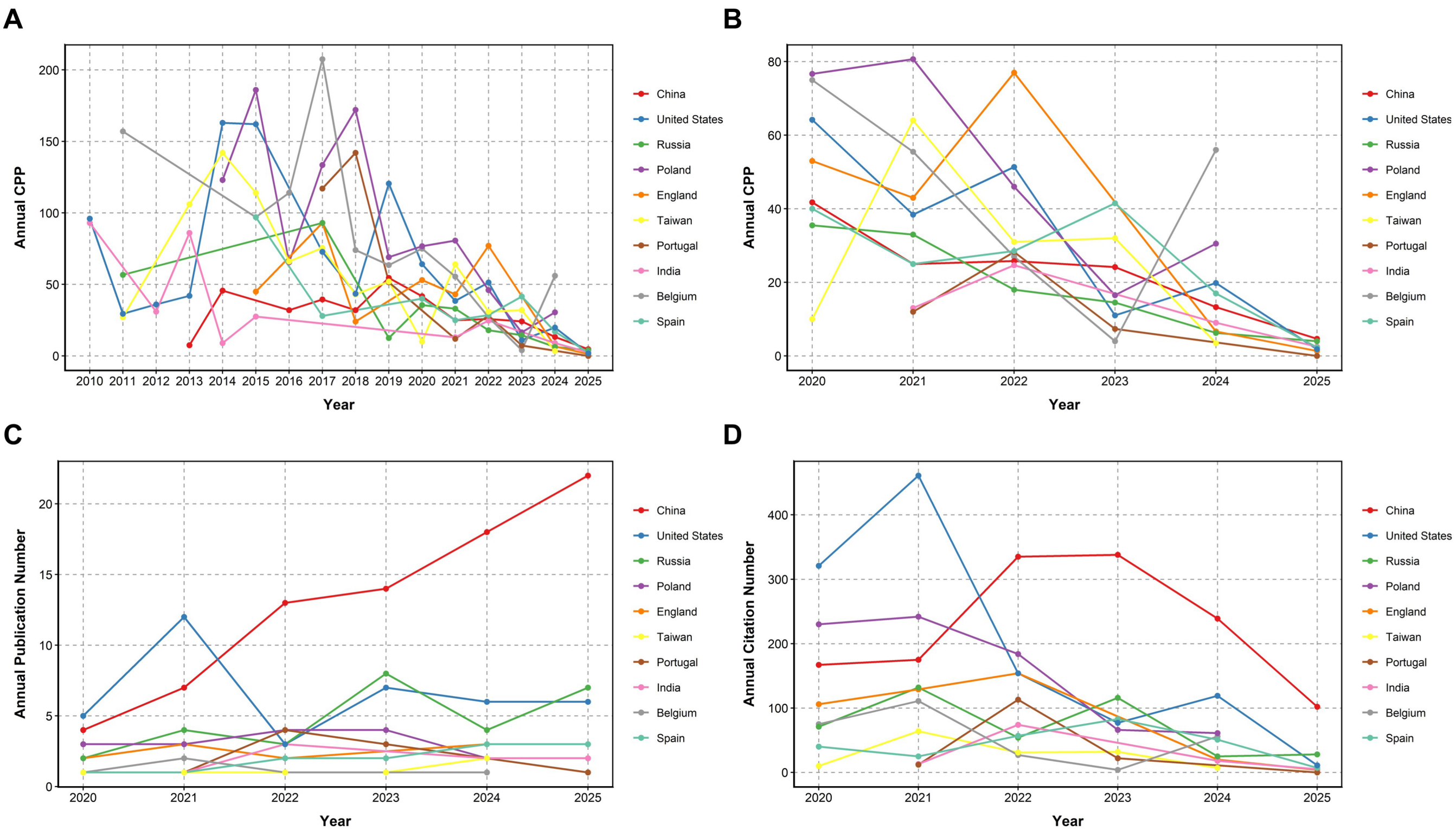

Figure S2. Annual publication performance of the top 10 countries/regions. (A) CPP, 2010–2025. (B) annual citations per publication (CPP), 2020–2025. (C) Publication output, 2020–2025. (D) Citation counts, 2020–2025.

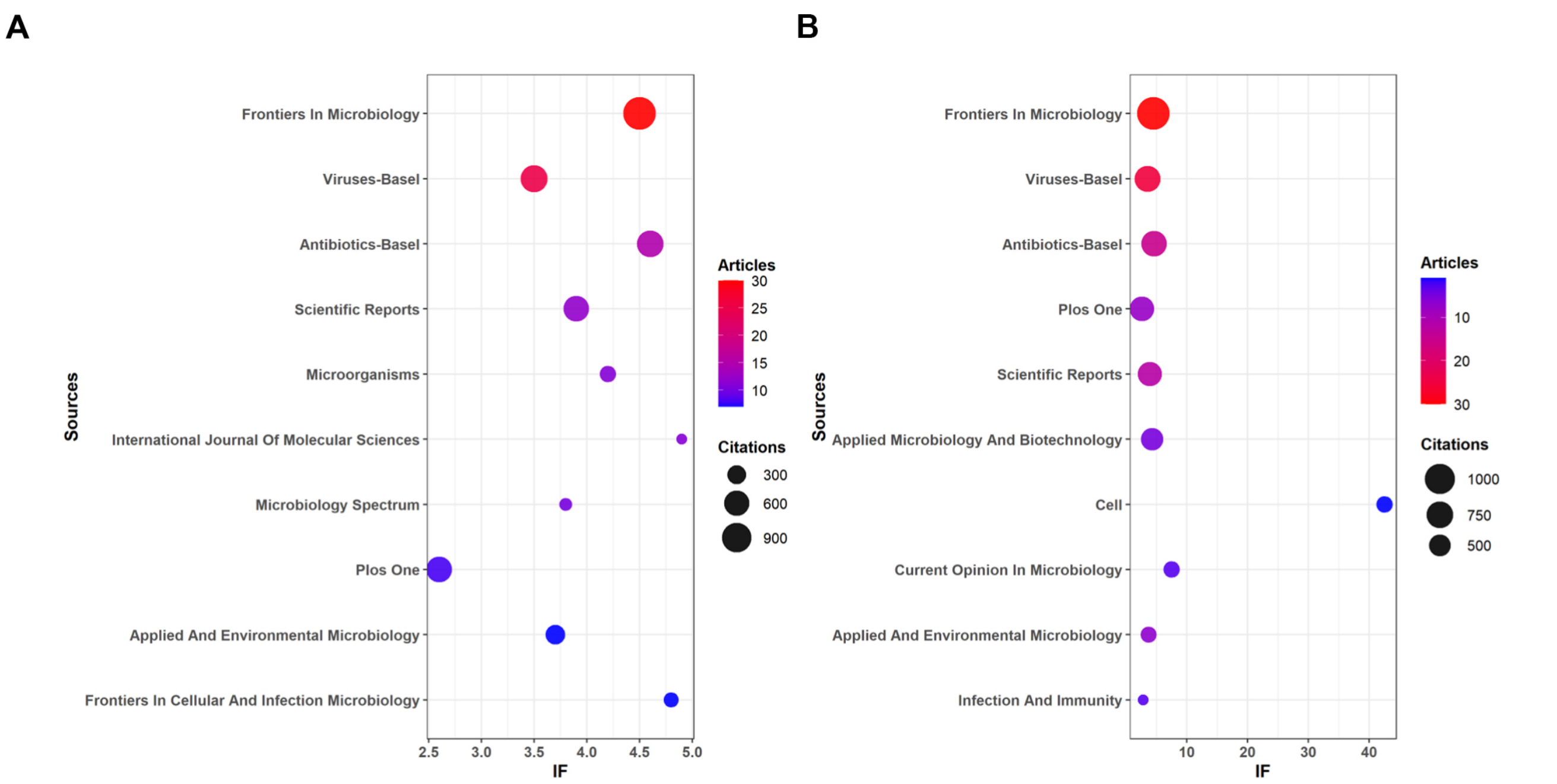

Figure S3. Source journal in phage depolymerase-related anti-infective research with the most publications **(A)** and citations **(B)**. Bubble size represents the number of citations, and color represents the number of articles.
